# Understanding the transition to viable but non-culturable state in *Escherichia coli* W3110: a comprehensive analysis of potential spectrochemical biomarkers

**DOI:** 10.1007/s11274-024-04019-6

**Published:** 2024-05-16

**Authors:** Özge Kaygusuz İzgördü, Rafig Gurbanov, Cihan Darcan

**Affiliations:** 1 Biotechnology Application and Research Center, Bilecik Şeyh Edebali University, Bilecik, Turkey; 2 Department of Molecular Biology and Genetics, Institute of Graduate Education, Bilecik Şeyh Edebali University, Bilecik, Turkey; 3 Department of Bioengineering, Bilecik Şeyh Edebali University, Bilecik, Turkey; 4 Central Research Laboratory, Bilecik Şeyh Edebali University, Bilecik, Turkey; 5 Department of Molecular Biology and Genetics, Bilecik Şeyh Edebali University, Bilecik, Turkey

**Keywords:** *E. coli* W3110, Viable but non-culturable (VBNC), Infrared spectroscopy, Copper, Erythromycin

## Abstract

The viable but non-culturable (VBNC) state is considered a survival strategy employed by bacteria to endure stressful conditions, allowing them to stay alive. Bacteria in this state remain unnoticed in live cell counts as they cannot proliferate in standard culture media. VBNC cells pose a significant health risk because they retain their virulence and can revive when conditions normalize. Hence, it is crucial to develop fast, reliable, and cost-effective methods to detect bacteria in the VBNC state, particularly in the context of public health, food safety, and microbial control assessments. This research examined the biomolecular changes in *Escherichia coli* W3110 induced into the VBNC state in artificial seawater under three different stress conditions (temperature, metal, and antibiotic). Initially, confirmation of VBNC cells under various stresses was done using fluorescence microscopy and plate counts. Subsequently, lipid peroxidation was assessed through the TBARS assay, revealing a notable increase in peroxidation end-products in VBNC cells compared to controls. ATR-FTIR spectroscopy and chemomometrics were employed to analyze biomolecular changes, uncovering significant spectral differences in RNA, protein, and nucleic acid concentrations in VBNC cells compared to controls. Notably, RNA levels increased, while protein and nucleic acid amounts decreased. ROC analyses identified the 995 cm^− 1^ RNA band as a consistent marker across all studied stress conditions, suggesting its potential as a robust biomarker for detecting cells induced into the VBNC state under various stressors.

## Introduction

Microorganisms are exposed to a wide variety of environmental stresses such as nutrient deficiency, temperature, osmotic stress, suboptimal pH, high oxygen concentration, heavy metals, inorganic salts, and antibiotics (Matilla 2018; Jia et al. 2020; Pazos-Rojas et al. 2023). When exposed to these stresses or adapted to unfavorable conditions, they enter a state known as Viable But Non-Culturable (VBNC) to survive (Sachidanandham et al. 2009; Yamamoto 2000). This state means that microorganisms lose their ability to reproduce and cannot be produced by conventional culture methods. Cells in this state have an intact membrane, undamaged genetic material, and cytoplasmic membrane. Therefore, these microorganisms are still alive and continue to perform various molecular, physiological, and metabolic activities (Kim et al. 2018; Ayrapetyan et al. 2018; Dong et al. 2020; Yoon and Lee 2020; İzgördü et al. 2022). In addition, there are various changes in cells in the VBNC state compared with culturable cells. Previous studies demonstrated that cell wall structure (Signoretto et al. 2000) and cell morphology (Takeda 2011) change in VBNC cells, and a decrease in cell size occurs (Ye et al. 2020). It was determined that the rod-shaped cells change to a coke and similar form and the spiral-shaped cells change to a spherical form when they switch to the VBNC state. These morphological changes are closely related to changes in cell wall components such as fatty acid composition, peptidoglycan cross-links, lipoprotein, and glycan chains in cytoplasmic membranes (Ding et al. 2017; Zhao et al. 2017; Dong et al. 2020; Progulske-Fox et al. 2022). Moreover, cells in the VBNC state showed increased peptidoglycan synthesis. Because of increased peptidoglycan synthesis and cross-linking, VBNC cells have much higher resistance to physical and chemical factors than culturable cells (Mederma et al. 1992; González-Escalona et al. 2006; Hung et al. 2013; Robben et al. 2018, 2019; Fu et al. 2020; Borkar et al. 2024). In addition, there are several physiological and molecular differences between cells in the VBNC state and culturable cells, including virulence potential, metabolism, ATP synthesis, gene expression, RNA quantity, and protein profiles (Mederma et al. 1992; González-Escalona et al. 2006; Hung et al. 2013; Robben et al. 2018, 2019; Fu et al. 2020).

When environmental conditions become favorable again or specific treatments are applied, cells in the VBNC state have the capability to transition back to an active and culturable state. This process is referred to as resuscitation (Zeng et al. 2013; Wei and Zhao 2018; Dong et al. 2020; Fu et al. 2020; Xu et al. 2022). To date, VBNC status has been detected in more than 100 microbial species from food, air, soil, and water, including *Escherichia coli, Vibrio cholerae, Staphylococcus aureus, Micrococcus luteus, Cronobacter sakazakii, Lactobacillus brevis, Campylobacter jejuni, Mycobacterium tuberculosis*, and *Klebsiella pneumoniae* (Oliver 2005, 2010, 2016; Pinto et al. 2015; Zhao et al. 2017; Dong et al. 2020; Ye et al. 2020; Ou et al. 2021). Studies have revealed that bacterial species retain their pathogenic properties in the VBNC state (Cervero-Aragó et al. 2019; Zhong and Zhao 2019). VBNC cells pose a significant public health problem because they can maintain their pathogenic properties, reactivate under favorable conditions, and cannot be detected by conventional culture methods (Ravel et al. 1995; Dinu and Bach 2013). Thus, it is critical to develop and use appropriate techniques for the detection of VBNC bacteria for the determination of microbial contamination levels in various environments, such as food, hospitals, and water sources (Ramamurthy et al. 2014; Zhang et al. 2023; Borkar et al. 2024).

Various viability measurement methods have been developed for the detection of VBNC cells. The first of these methods is direct viable count (DVC), which is a microscopic technique (Kogure et al. 1979). In this method, cells in the VBNC state are treated with nalidixic acid and then stained with acridine orange and counted directly under the microscope (Kogure et al. 1979; Xu et al. 1982; Zhang et al. 2023). Another method, CTC-DAPI staining is used to determine cellular activity in VBNC cells. In this method, the metabolic activity of cells is determined using a combination of fluorescent components such as CTC (5-cyano-2,3-di-(p-tolyl) tetrazolium klorid) and DAPI (4’,6-diamidino-2-phenylindole) (Severin et al. 1985; Fakruddin et al. 2013; Wideman et al. 2021). Another method used in the detection of VBNC cells is the assessment of cytoplasmic membrane integrity. In this approach, two different fluorescent dyes, nucleic acid-based SYTO-9 and propidium iodide, are used together. SYTO-9 produces green fluorescence by staining the nucleic acids of living cells, whereas propidium iodide produces red fluorescence by staining the nucleic acids of cells, particularly those with membrane damage. This combination is used to differentiate cells in the VBNC state into live and dead cells by assessing cytoplasmic membrane integrity (Zhao et al. 2013; Orman and Brynildsen 2013; Park and Kim 2018; Song and Lee 2021; Zhou et al. 2022; Zhang et al. 2023). As an alternative to staining-based methods, molecular biology techniques such as PMA-qPCR (Propidium Monoazide-quantitative Polymerase Chain Reaction) and RT-qPCR (Reverse Transcription-quantitative Polymerase Chain Reaction) have also been developed. PMA-qPCR offers a nucleic acid-based approach to distinguish live and dead cells, while RT-qPCR uses actively expressed housekeeping and virulence genes as viability markers. As an alternative to these molecular methods, uncommon methods such as Raman spectroscopy, Maldi-Tof-Ms, and ATP-luciferase assay are also used to detect VBNC cells (Nocker et al. 2006; Nocker and Camper 2009; Gin and Goh 2013; Zhang et al. 2015; Ding et al. 2017; Zhong and Zhao 2018; Wideman et al. 2021).

The above mentioned methods are the gold standard techniques used to detect VBNC cells. However, the need for new techniques that will enable rapid, practical, and cost-effective detection of VBNC cells to ensure public health and food safety cannot be ignored. Therefore, researchers continue their search to develop new techniques. Fourier Transform Infrared (FTIR) Spectroscopy technology, which is commonly used to detect microorganisms at the species level, attracts attention in this context. FTIR spectroscopy is a technique introduced in 1991 for the identification and classification of microorganisms. Since then, this method has received increasing attention and has undergone remarkable evolution. These advances have led to FTIR spectroscopy being accepted as an important research and diagnostic method in microbiology. Today, this technique provides fingerprint spectra in bacterial isolates that allow rapid characterization of microbial strains and enable the identification of microorganisms at the species level, making it possible to examine complex microbial samples quickly and reliably. Additionally, the technique is capable of detecting changes in the biochemical components of microorganisms (Schmitt and Flemming 1998; Erukhimovitch et al. 2005; Wenning and Scherer 2013). From this perspective, ATR-FTIR spectroscopy offers a potential solution in the of detection of VBNC cells. Biochemical properties of VBNC cells can also be examined and detected. In ATR-FTIR spectral analyses performed on cells induced into the VBNC state, significant spectral modifications were detected in many microorganism species that entered the VBNC state (Jia et al. 2020; Niedźwiedź et al. 2020; Xie et al. 2021).

This study aims to determine the biomolecular changes occurring in *Escherichia coli* cells induced into a Viable but Non-Culturable (VBNC) state under different stress conditions such as heat, heavy metal, and antibiotic, by combining ATR-FTIR spectroscopy with unsupervised chemometric approach, principal component analysis (PCA). The VBNC state of bacteria was also examined by microbiological and biochemical assays and fluorescence staining. Specifically, the study focuses on examining the spectrochemical biomarkers in bacteria associated with the transition to the VBNC state.

## Materials and methods

### Bacterial strain and growth conditions

*Escherichia coli (E. coli)* strain W3110 was used in this study. This strain was stored at -80 °C in LB broth containing glycerol. At the beginning of the study, cells were removed from − 80 °C, inoculated onto LB agar plates to form a single colony, and incubated at 37 °C for 24 h. A single colony selected from the cells growing on Petri plates was transferred to LB broth liquid medium and incubated under the same conditions at a shaking speed of 160 rpm until reaching the exponential phase (OD_600_ = 1). Cells obtained at this stage were used in life experiments in artificial seawater (Bogosian et al. 1996; Rigsbee et al. 1997; Na et al. 2006; Darcan et al. 2009; Idil et al. 2010; Xia et al. 2017; Castro-Rosas et al. 2017; Casasola-Rodríguez et al. 2018).

### Induction of VBNC cells

Artificial seawater was prepared for the life experiments. The artificial seawater to be used in the study was prepared according to Rigsbee et al. (1997) to contain 24.723 g NaCl, 4.668 g MgCl_2_, 6.298 g MgSO_4_, 1.36 g CaCl_2_, 0.67 g KCl, and 0.18 g NaHCO_3_ per liter and distributed into 50 ml flasks and sterilized by autoclaving (15 min, 121◦C) (Rigsbee et al. 1997).

Cells grown to exponential phase to conduct survival experiments in the presence of temperature stress were washed twice with 1X PBS, resuspended in 50 ml artificial seawater, and incubated statically at 42 °C until 6 days when the plate count was zero.

To perform survival experiments in the presence of antibiotic stress, erythromycin at 937.5 µg/ml was added to 50 ml of artificial seawater. Following the addition of the antibiotic, the cells were washed with 1X PBS, resuspended in 50 ml of artificial seawater, and the flasks were incubated stationary at 25 °C for 40 days until the plate count reached zero.

Copper sulfate (CuSO_4_) was used as metal stress in the study. For this purpose, CuSO_4_ was added to 50 ml of artificial seawater with a final concentration of 20 µM. Then, the cells were washed with 1X PBS, followed by resuspension in 50 ml of artificial seawater, and incubated stationary at 25 °C for up to 8 days until the plate count reached zero.

The cells that were not exposed to stress and incubated in artificial seawater at 25 °C for the same period as the stress conditions were used as the control group for all stress conditions. Then, samples were taken from these bottles every 2 days for temperature and metal stress and every 10 days for antibiotic stress, and the transition of the cells to the VBNC state was observed. With the samples taken, bacteria that could form colonies were counted (by plate counting), and bacteria with membrane integrity were counted (Live/Dead BacLight kit). Control group plate and live dead counts were performed simultaneously under stress conditions.

### Analysis of viability and culturability

From life experiments conducted under different stress conditions, 100 µl samples were taken at determined time intervals, a 1:10 serial dilution was made in 1X PBS, and the samples were placed in Petri dishes containing nutrient agar with a Drigalski spatula. The Petri dishes were incubated at 37 °C, and cell numbers were calculated as numbers per milliliter (CFU/mL) at the end of 24 h. In addition, the Petri dishes were kept at room temperature for another 48 h and counted again, and it was checked whether there was any change in the previous counts (Masmoudi et al. 2010; Orman and Brynildsen 2013; Guo et al. 2019; Jia et al. 2020; Xie et al. 2021).

While samples were taken for plate counting, dead live cells were also counted with the Live/Dead BacLight kit (Zhao et al. 2013; Park and Kim 2018; Song and Lee 2021; Zhou et al. 2022; Zhang et al. 2023). Total and viable cell numbers in the samples were determined by staining with the Live/Dead BacLight bacterial viability kit. This kit contains a mixture of two nucleic acid dyes, SYTO 9 and propodium iodide (PI), to assess cell membrane integrity. SYTO 9 (yields green fluorescence) can penetrate the plasma membrane of both intact and damaged bacterial cells, whereas PI (yields red fluorescence) can only penetrate cells with damaged plasma membranes and compete with SYTO 9 for nucleic acid binding sites. Therefore, cells with intact cell membranes (thought to be alive) show green fluorescence, whereas cells with damaged membranes (thought to be dead) show red fluorescence. Before the experiment, two dyes were prepared by mixing them in a 1:1 ratio. One milliliter of the artificial seawater samples to be counted was taken into tubes and 3 µl of the Live/Dead mixture was added. After the mixture was left in the dark at room temperature for 15 min, the cells were imaged under a fluorescence microscope. At least 20 different areas were counted, and the average of these areas was taken to reach the number of bacteria per ml from the microscope area.

### Lipid peroxidation analysis

Cells that were confirmed to be inducted into the VBNC state by plate count results and the Live/Dead Baclight kit were collected using a vacuum filtration system with a 47 mm wide polycarbonate filter with 0.2 μm pore diameter. Both VBNC and control cells collected with filters were washed once with 1X PBS, redissolved in 300 µl PBS, and 300 µl 20% TCA was added and incubated at -20 °C for 20 min. After incubation, all impurities were removed by centrifugation. After centrifugation, 250 µl of supernatant was transferred to a clean tube and 600 µl of 0.67% TBA was added and heated at 100 °C for 30 min. Following this process, the samples were cooled on ice for 15 min, and readings were taken on the spectrophotometer at wavelengths of 532 and 600 nm for the measurement of malondialdehyde (MDA) levels. Calibration coefficient ε = 1.56 × 10^5^ M^− 1^ cm^− 1^ (Dolezalova and Lukes 2015; Borkar et al. 2024).

### Infrared spectral analyses

Bacterial cells induced into a VBNC state under three different stress conditions were collected using a vacuum filtration method with a 47 mm wide polycarbonate filter with a pore size of 0.2 μm. Subsequently, these cells were washed through the filter with dH_2_O and centrifuged at 12,000 g for 10 min. Then, the supernatant was removed, and the pellet was washed again with 1 ml dH_2_O and centrifuged at 12,000 g for 10 min. Following this, the cell pellet was suspended in 100 µl dH_2_O, and 1 µl was dropped onto a Diamond/ZnSe crystal plate of ATR-FTIR Spectrometer (Perkin-Elmer, US) and dried under a gentle nitrogen flow for 2 min. Subsequently, it was scanned in the spectral range of 4000 to 650 cm^− 1^ at room temperature with a resolution of 4 cm^− 1^ and 32 scans (Gurbanov et al. 2021; Kilicaslan et al. 2023).

Quantitative spectral data analysis was performed using OPUS 5.5 (Bruker) software. The spectra of each sample were baseline corrected using the Rubberband correction method with 64 baseline points excluding CO_2_ bands. In a detailed quantitative spectrochemical analysis, the bands with the highest absorbance values in different spectral regions of the spectra were selected, and the bands’ initial and latest frequencies were determined with precision. The areas of bands specific to various biomolecules were analyzed by taking the integral areas of the determined frequency ranges with the OPUS 5.5 (Bruker) software (Ardahanlı et al. 2022; Ceylani et al. 2022).

### Chemometric analysis

PCA was administered to differentiate the spectral data of different bacterial groups from each other and estimate the responsible variables for VBNC state. The spectral transformations are valuable to make the qualitative and quantitative analyses as independent as possible from the FTIR spectrometers. Hence, two different transformations were applied to raw spectra before the analysis. First, the baseline transform was done using the baseline offset method over 4000 –650 cm^− 1^ spectral region. Second, Unit Vector Normalization was employed over the same region. The mean-centered spectral data were cross-validated in data mining using a full method with nine segments. The number of calibration samples was 9. The matrix factorization method, the singular value decomposition (SVD) algorithm, was applied in the spectral range of 1300 –700 cm^− 1^. The maximum number of principal components was 7. The results were given as scores and loadings plots.

### Statistical analysis

The analyses were performed using GraphPad Prism 8.01 (GraphPad Software, San Diego, California), a statistical analysis program. Data was assessed using Tukey’s multiple comparisons test within the framework of ordinary One-way ANOVA. The degree of significance was always set at a 95% confidence interval and denoted as less than or equal to *P* < 0.05 *, *P* < 0.01 **, *P* < 0.001 ***, and *P* < 0.0001 ****. The results were expressed as means ± standard error of the mean.

Receiver Operating Characteristic (ROC) analyses were also conducted using GraphPad Prism 8.01 software, in which the area under the curve (AUC) and P values were computed at a 95% confidence interval. The % points of sensitivity (True Positive Rate) and specificity (False Positive Rate) were plotted on an ROC curve graph as the threshold varies.

## Results

### Induction of VBNC state, viability detection, and TBARS analysis in *E. Coli* W3110

**Fig. 1:**
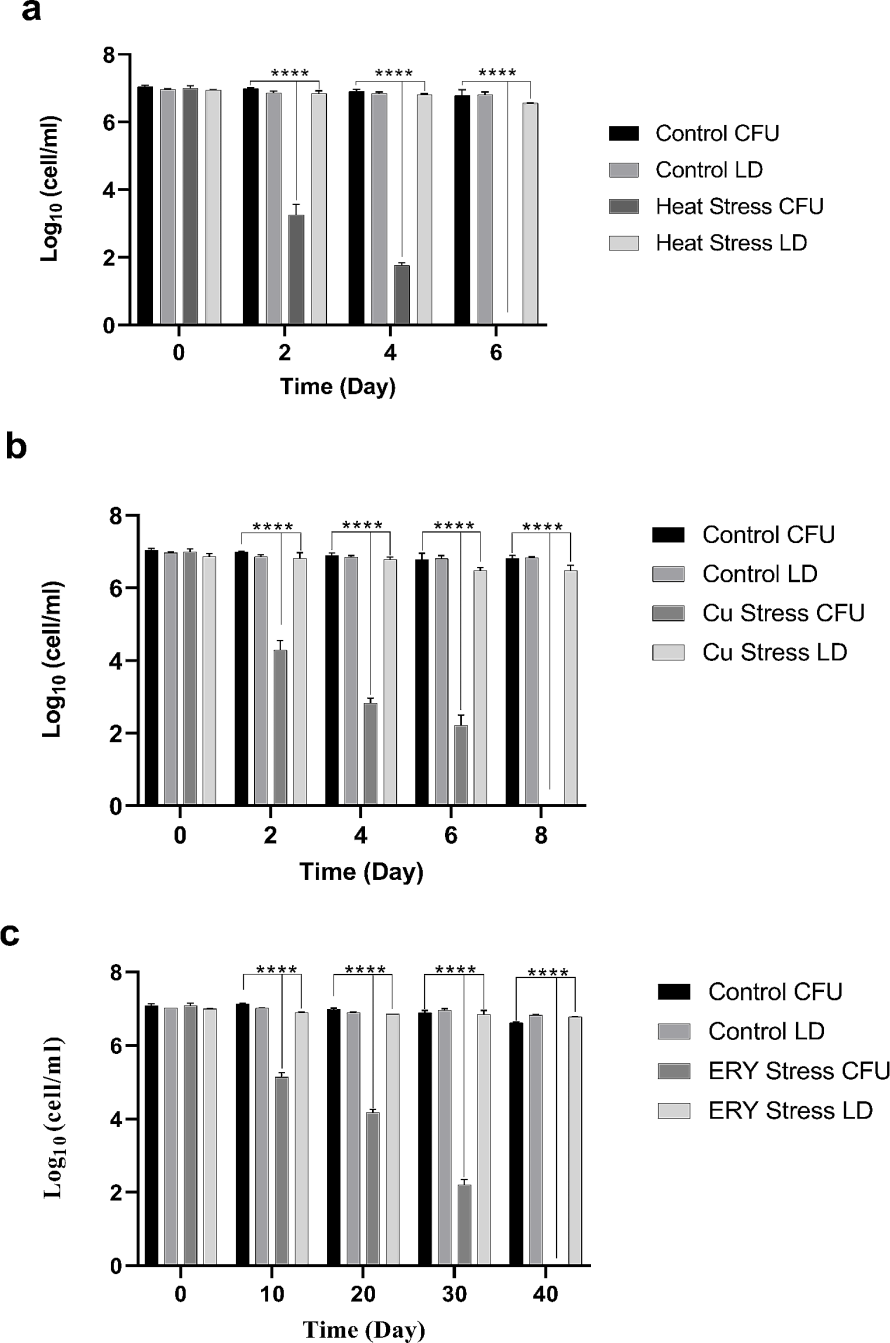
Survival experiment results for *Escherichia coli* W3110 induced into VBNC state under different stress conditions. *Escherichia coli* under **(a)** heat, **(b)** copper (heavy metal), and **(c)** erythromycin (antibiotic) stress conditions. The degree of significance was denoted as less than or equal to *P* < 0.0001 ****. The results were expressed as means ± standard error of the mean (CFU: Colony forming unit, LD: Live/Dead BacLight count)

**Fig. 2:**
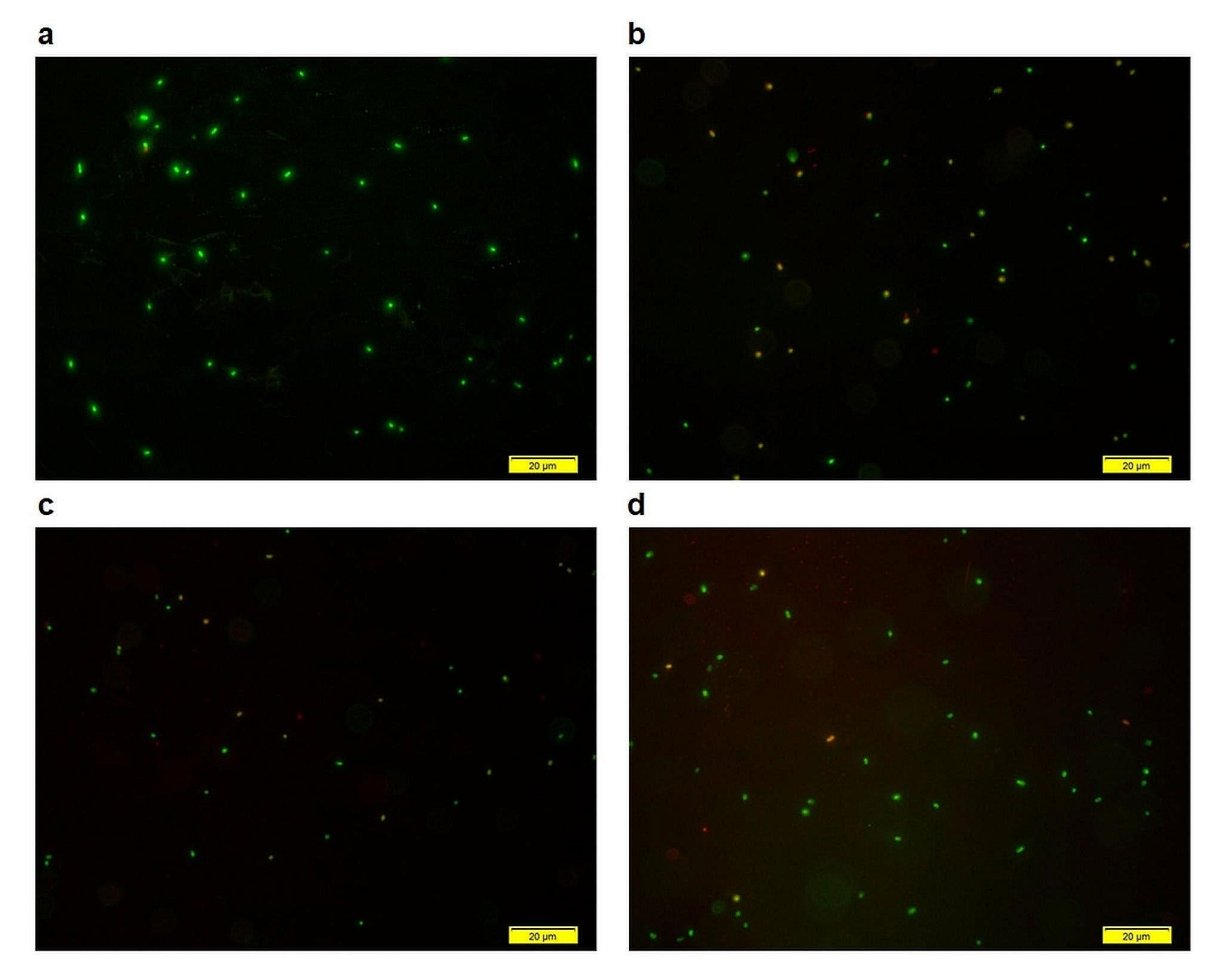
Images of VBNC cells under a fluorescent microscope. Cells were stained with the LIVE/DEAD BacLight Kit to differentiate living cells (green) and dead cells (red). Treatments include **(a)** control, **(b)** heat, **(c)** copper (heavy metal), and **(d)** erythromycin (antibiotic). The scale bar represents 20 μm

**Fig. 3:**
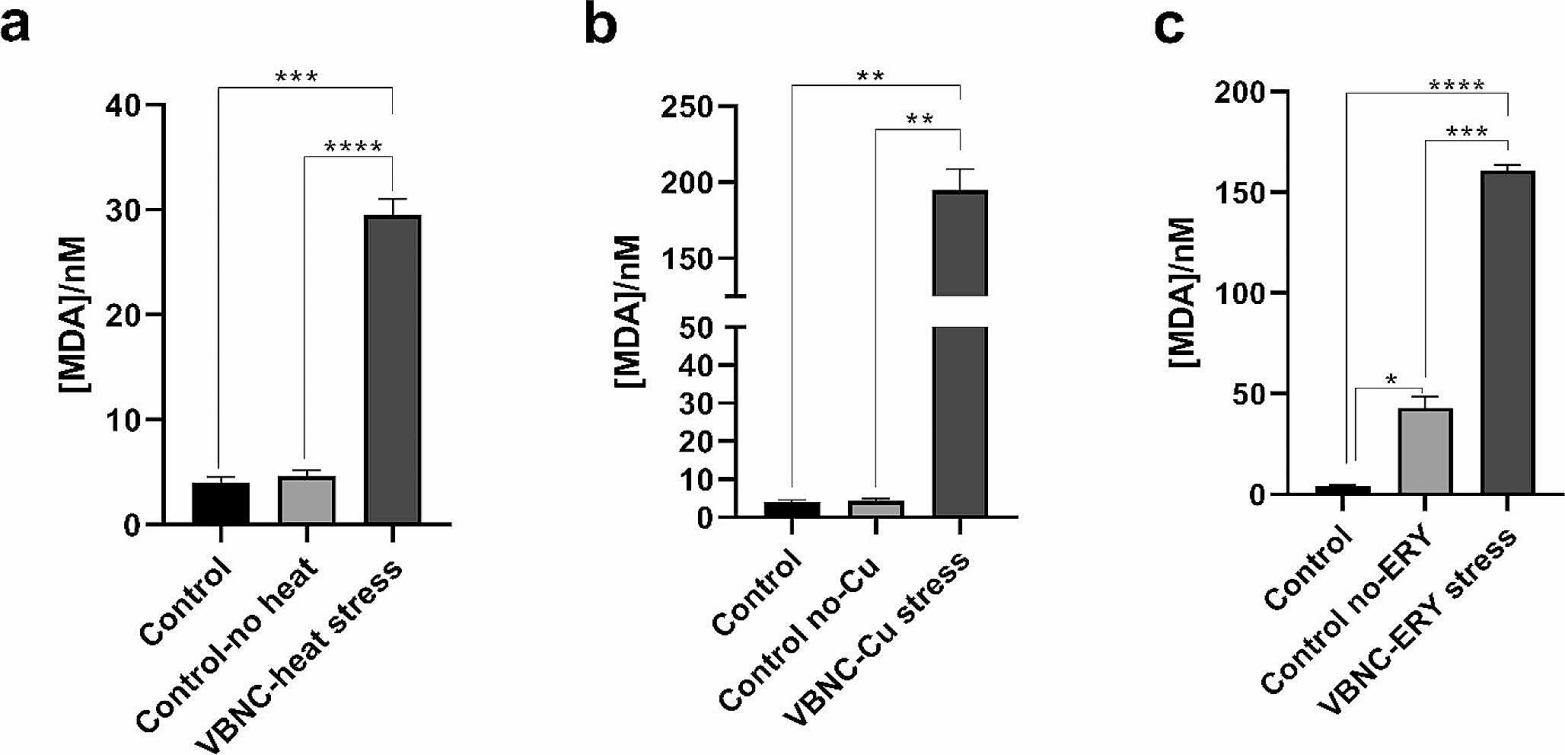
Lipid peroxidation (malondialdehyde/ MDA) levels in *Escherichia coli* W3110 in the VBNC state under **(a)** heat, **(b)** copper (heavy metal), and **(c)** erythromycin (antibiotic) stress conditions. The degree of significance was denoted as less than or equal to *P* < 0.05 *, *P* < 0.01 **, *P* < 0.001 ***, and *P* < 0.0001 ****. The results were expressed as means ± standard error of the mean

The main purpose of this study was to analyze the biomolecular changes occurring in *E. coli* W3110 induced by the VBNC state under the influence of environmental factors such as heat stress, metal presence, and antibiotic exposure. For this purpose, *E. coli* W3110 cells were first induced into the VBNC state under all stress factors, and the induction of the cells into the VBNC state was confirmed by plate count and Live/Dead count results (Figs. 1 and 2). In artificial seawater conditions, the plate counts of *E. coli* cells dropped to zero at 6, 8, and 40 days under temperature, metal, and antibiotic stresses compared with the control group cells (Fig. 1). However, simultaneous Live/Dead BacLight counting results indicated that cell viability was still ongoing, and the cells had entered the VBNC state (Fig. 2). The transition of microorganisms to the VBNC state may cause changes in cell lipid structures and amounts under the influence of various stress factors. In this context, the MDA quantities in the cells transitioning to the VBNC state under different stress conditions, such as temperature, metal, and antibiotics, were also measured using TBARS assay. VBNC cells exhibited higher levels of lipid peroxidation compared to control cells under three different stresses. Notably, copper and antibiotic stresses caused a greater increase in peroxidation compared to heat stress (Fig. 3).

### Qualitative chemometric findings

Infrared (IR) spectroscopy is a powerful analytical technique used to study the vibrational transitions of molecules. It works by irradiating a sample with infrared light and measuring the absorption or transmission of the light at different frequencies. This absorption spectrum provides information about the functional groups present in the molecules, as different chemical bonds absorb infrared radiation at specific frequencies. By analyzing the bands and patterns in the IR spectrum, one can identify the types of chemical bonds present in the sample and infer the molecular structure. IR spectroscopy is widely used in various fields such as chemistry, biochemistry, biology, and microbiology for qualitative and quantitative analysis of organic and inorganic compounds. In addition, IR spectroscopy is used in the analysis of cellular components such as carbohydrates, lipids, proteins, and nucleic acids (Stuart 2005; Baker et al. 2014; Othman and Othman 2022; Tiquia-Arashiro et al. 2023).

The main scope of the study is to reveal the biomolecular modulations in *E. coli* W3110 during the VBNC state, which is induced by various stress factors such as heat, copper (heavy metal), and erythromycin (antibiotic) via IR spectroscopy. Hence, the raw IR spectral data were accordingly transformed and subsequently analyzed using a chemometric data mining technique - unsupervised PCA, to explicitly determine the critical spectral locations of major affected functional groups in bacterial biomolecules that is spectrochemical bands.

**Fig. 4:**
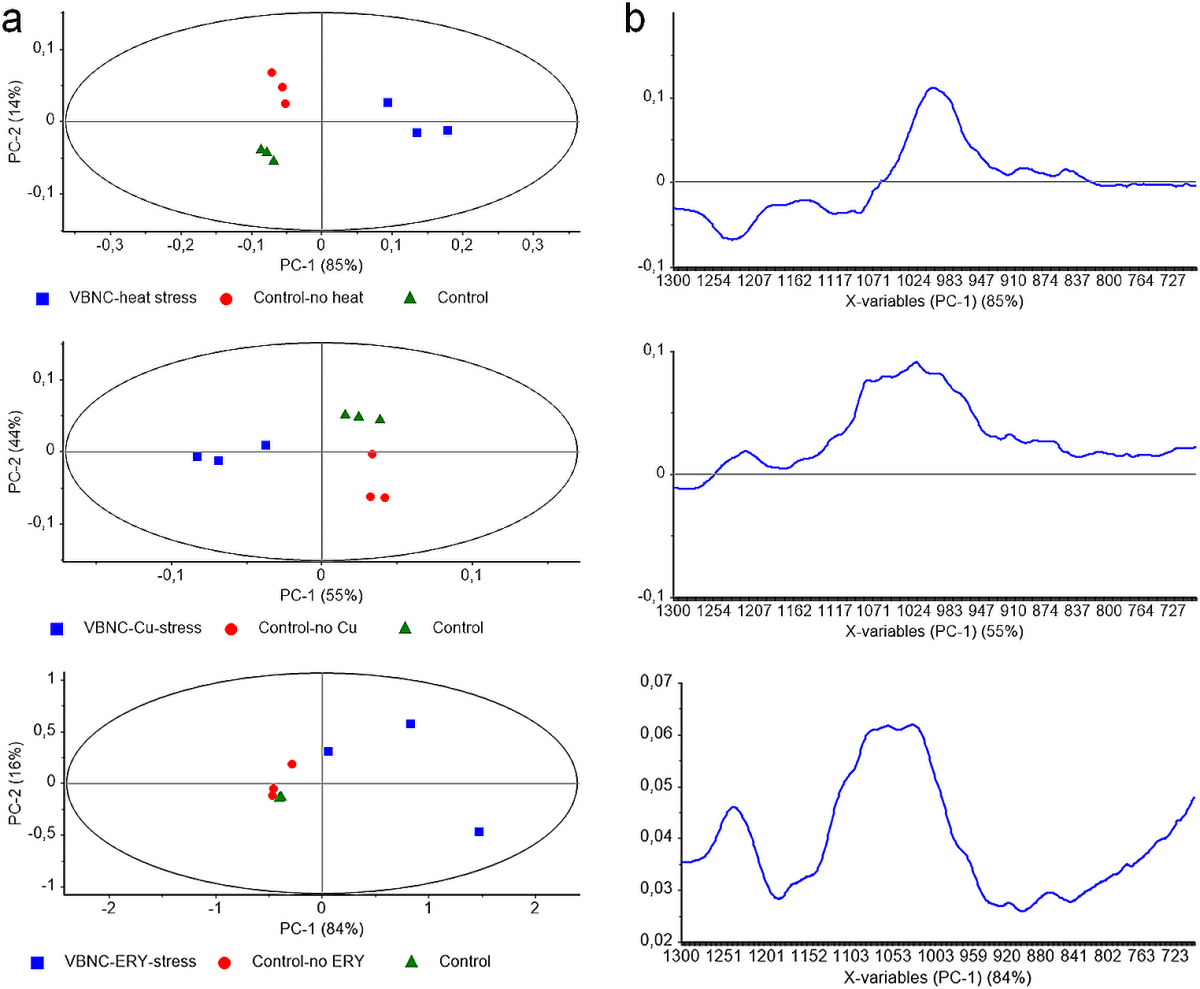
Principal Component Analysis illustrating biomolecular events associated with VBNC state in *Escherichia coli* W3110. **(a)** Graphs depicting scores and **(b)** loadings plots show VBNC induced by heat, copper (heavy metal), and erythromycin (antibiotic) stresses, alongside experimental control and independent control samples within the 1300 –700 cm^-1^ spectral range

The structural and functional changes of biomolecules in cells induced into the VBNC state were compared with those in control group cells using IR spectroscopy coupled with chemometrics. The analyses revealed significant spectral differences in cells induced into the VBNC state compared with the control group cells. During the general chemometric evaluations; the VBNC bacteria were differentiated from experimental and independent controls and this event was valid for different stress factors (Fig. 4). Technically speaking, the differentiation percentages of VBNC bacteria (shown as blue squares on the scores plots) from control ones were revealed in the range of 55–85% across the PC-1 axis of scores plots. Independent controls and experimental controls were also separated from each other in the range of 14–44% across the PC-2 plane (Fig. 4a, left panel). As a rule of thumb, PC-1 and PC-2 are major principal components that are examined during the evaluation of gross changes, in contrast to other PCs that are considered during the evaluation of small alterations. The loadings plots demonstrated the approximate locations of spectral changes (discriminators) responsible for these differentiations across the same axis (Fig. 4b, right panel).

**Fig. 5:**
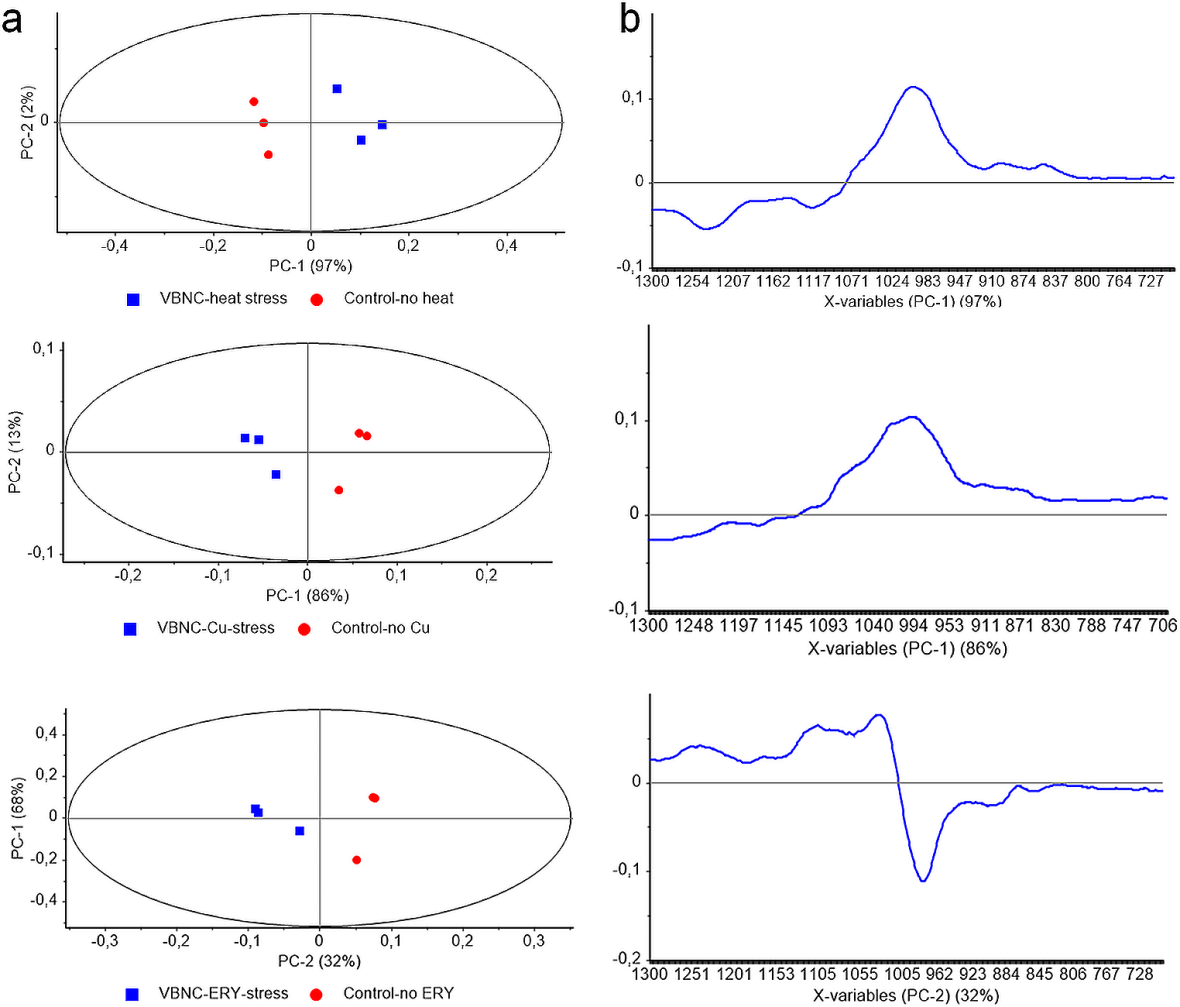
Principal Component Analysis illustrating biomolecular events associated with VBNC state in *Escherichia coli* W3110. **(a)** Graphs depicting scores and **(b)** loadings plots show VBNC induced by heat, copper (heavy metal), and erythromycin (antibiotic) stresses, alongside experimental control samples within the 1300 –700 cm^-1^ spectral range

To ensure the precise locations of spectral discriminators, PCA was rerun, this time involving VBNC and experimental control bacteria, excluding independent control bacteria (Fig. 5). This scenario revealed more precise results; the VBNC bacteria were differentiated from experimental control bacteria across the PC-1 planes with 97% and 86% for heat and copper stresses, respectively. On the other hand, the most effective differentiation was obtained across the PC-2 plane for erythromycin-induced VBNC cells with 32% (Fig. 5a, left panel). The evaluation of loadings plots revealed precise locations of spectral discriminators standing behind the observed differentiation. For heat and copper stresses the positive discriminator at 995 cm^-1^, and for erythromycin, the negative discriminator at the same spectral location strongly pinpointed the VBNC-associated modulation in *E. coli* W3110 (Fig. 5b, right panel).

### Quantitative spectrochemical findings

**Fig. 6:**
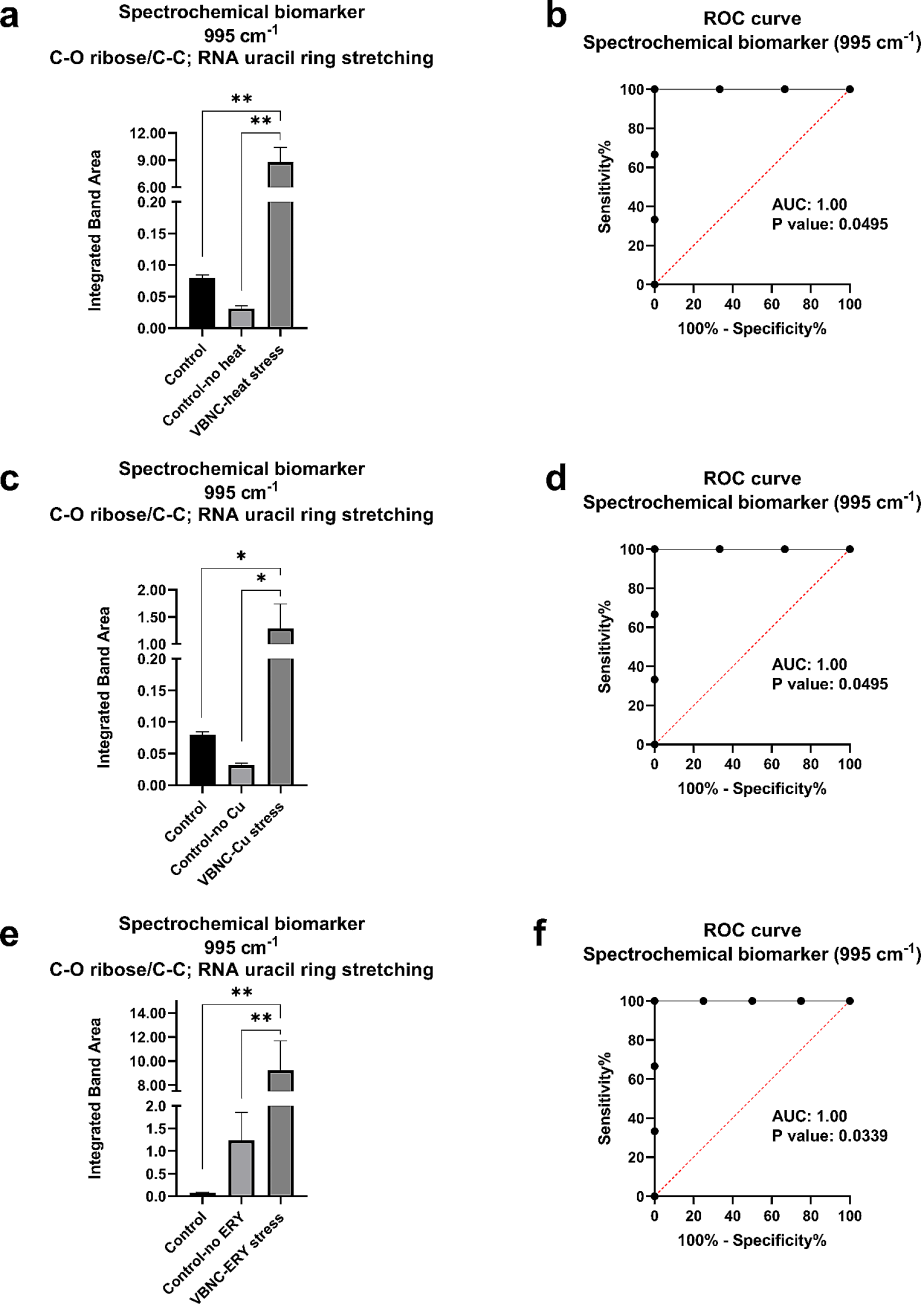
Quantification and validation of spectrochemical biomarker band for VBNC state in *Escherichia coli* W3110 under **(a-b)** heat, **(c-d)** copper, and **(e-f)** erythromycin stress conditions. The degree of significance was denoted as less than or equal to *P* < 0.05 * and *P* < 0.01 **. The results were expressed as means ± standard error of the mean

Chemometric-assisted investigation of VBNC-related modulations helped to identify the major spectrochemical parameters that were further quantitated using the band integration method explained in the data analysis section. For validation purposes, the quantitative analyses were also supported by ROC analyses. As reported above, the major spectrochemical marker associated with the VBNC state was the band emerging around 995 cm^− 1^ spectral position. Hence, the study especially focused on the quantification and validation of this band which can be potentially used as a reliable biomarker in fast track of VBNC status in laboratory conditions. According to the literature, the proposed VBNC biomarker band at 995 cm^− 1^ emerges from C-O ribose, C-C vibrations, and RNA uracil rings stretching vibrations (Ferreira et al. 2020). Generally, this band emerges from RNA molecules present in biological systems (Talari et al. 2017). The results show that concentrations of RNA band at 995 cm^− 1^ position significantly enhance in VBNC state for *E. coli* W3110 bacteria under all stress conditions compared with both independent control and experimental control bacteria. Validation of the results was done by ROC curves, which indicates noteworthy AUC values (1.00) under all stress conditions, implying that the RNA band can be considered a dependable spectrochemical biomarker for the VBNC state under laboratory settings (Fig. 6).

**Fig. 7:**
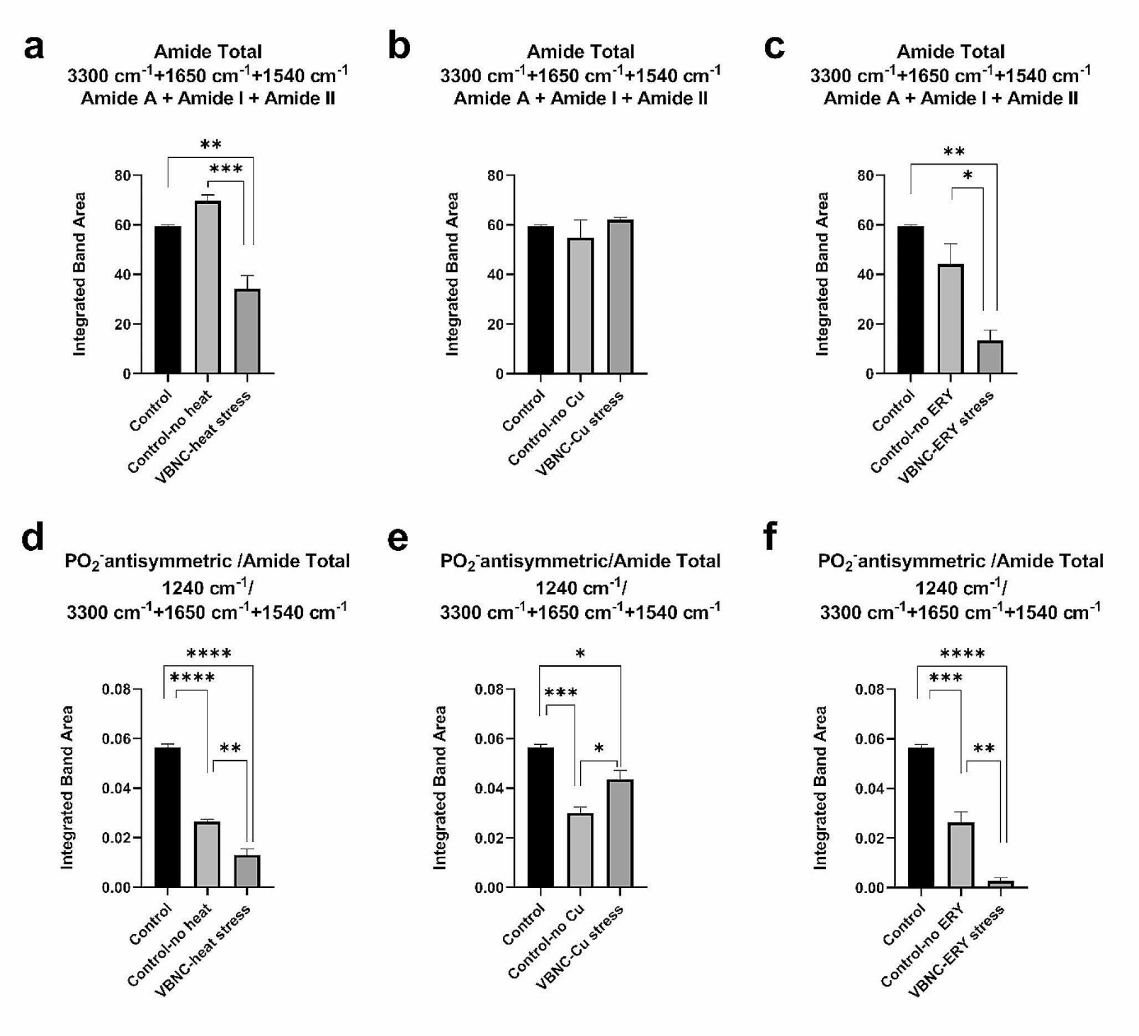
Quantification of various spectrochemical band indices for VBNC state in *Escherichia coli* W3110 under heat, copper, and erythromycin stress conditions. The changes in **(a-c)** Amide Total and **(d-f)** PO_2_ antisymmetric /Amide Total band ratios. The degree of significance was denoted as less than or equal to *P* < 0.05 *, *P* < 0.01 **, *P* < 0.001 ***, and *P* < 0.0001 ****. The results were expressed as means ± standard error of the mean

Other VBNC-associated biomolecular alterations were also quantified. The significant modulations happened in Amide Total and PO_2_ antisymmetric /Amide Total band area ratios, which are spectrochemical indices demonstrating total protein and nucleic acid quantitates, respectively (Yonar et al. 2022). Despite the substantial increase in RNA concentrations; VBNC bacteria have significantly reduced protein (Fig. 7a-c) and nucleic acid (Fig. 7d-f) amounts under heat and erythromycin stresses, except the copper stress conditions.

## Discussion

Under harsh environmental conditions, VBNC cells enter a “survival mode” to endure until favorable conditions return. This involves reducing their activity levels in functions like metabolism, gene production, and cell division. Consequently, these cells exhibit lower levels of proteins and nucleic acids, aligning with scientific reports documenting similar reductions in VBNC cells (Mederma et al. 1992; González-Escalona et al. 2006; Hung et al. 2013; Robben et al. 2018, 2019; Fu et al. 2020). Moreover, extensive modifications are observed in the peptidoglycan cross-links, the glycan chain, the lipoproteins, and the fatty acid composition of the cells in the VBNC state. These modifications provide the cells in the VBNC state with advantages for survival under stress conditions. However, membrane damage that occurs under stress conditions can be a possible reason for cells to switch to the VBNC state. When microorganisms in the VBNC state are exposed to environmental stressors, antioxidant defense mechanisms may weaken, leading to increased free radical formation and promoting lipid peroxidation. Numerous studies have found an increase in lipid peroxidation in cells in the VBNC state (Joshi et al. 2011; Dolezalova and Lukes 2015; Yost and Joshi 2015; Borkar et al. 2024). When MDA amounts were measured by the TBARS assay in cells induced into the VBNC state under different stress conditions, it was revealed that lipid peroxidation occurs under all three applied stress conditions. In particular, it was determined that MDA concentrations increase significantly in VBNC cells exposed to metal and antibiotic stresses. The reason for this increase may be that both the metal and the antibiotic increase the formation of free radicals, causing oxidative degradation of the cell membrane that leads to the formation of lipid peroxidation end products.

Infrared spectrochemical analyses performed in our study also confirmed most of the previous findings. In a study conducted with *E. coli* at 4 °C, it was found that the spectral bands at 2966 cm^− 1^, 2929 cm^− 1^, 2852 cm^− 1^ (lipid), 1637 cm^− 1^, 1545 cm^− 1^ (secondary structure of proteins), and 1235 cm^− 1^ (phosphodiesters) positions significantly decreased compared with the control samples. In the same study, the experiments conducted at -18 °C also revealed a decrease in the intensity of bands associated with lipids, nucleic acids, and structural proteins (Lu et al. 2011). After cold plasma treatment of *Lentilactobacillus hilgardii* cells, FTIR spectral analysis revealed significant spectral changes in VBNC cells compared with normal cells. Following plasma application, a decrease of bands around ~ 2960 cm^− 1^ and 2920 cm^− 1^, and a pronounced increase in bands around 1375 cm^− 1^, 1720 cm^− 1^, and 1045 cm^− 1^ were detected. These alterations were found to be associated with vibrations of functional groups belonging to fundamental cellular components such as fatty acids, carbohydrates, proteins, and nucleic acids (Niedźwiedź et al. 2020). Compared with normal cells, additional bands appeared in the infrared spectra of *Candida sp*. LN1 strain, induced to the VBNC state by high phenol concentration. Among the determined bands, there were differences in the bands at 2955 cm^− 1^ (methyl group asymmetric vibration band), 1539 cm^− 1^ (amide I and II bands of proteins), and 1044 cm^− 1^ associated with polysaccharides/nucleic acids. In addition, the absorption bands observed in the 1000 –900 cm^− 1^ spectral region were assigned to carbohydrates and polysaccharides in the cell wall, and these bands were considered to be indicators of VBNC status (Xie et al. 2021). Another study determined significant changes in the 1800 –1300 cm^− 1^ spectral region in *E. coli* ATCC 25922 and *Pseudomonas aeruginosa* ATCC 15442 cells exposed to different doses of free chlorine (0.0–1.0 ppm). In addition, PCA analysis revealed that cells exposed to chlorine and those not exposed to chlorine were separated from each other (Al-Qadiri et al. 2008). In a study conducted on *Campylobacter jejuni* induced into the VBNC state through variations in temperature and oxygen tension, Moen et al. (2005) observed a reduction in the proteins and an elevation in the polysaccharides (Moen et al. 2005). Similarly, the molecular structure of peptidoglycan in *Rhodococcus biphenylivorans* cells induced into a VBNC state in the presence of norfloxacin antibiotic was investigated using the ATR-FTIR spectroscopic method, revealing small yet significant differences in the 1200 –800 cm^− 1^ region (Jia et al. 2020).

The findings from our research, along with supporting evidence from existing literature, suggest that FTIR spectroscopy coupled with chemometric tools could be an applicable method to detect the presence of VBNC bacteria that are undetectable by traditional microbial techniques. Our study particularly emphasizes that the RNA band at 995 cm^− 1^ is an important result for the rapid and cost-effective detection of *E. coli* W3110 cells in the VBNC state. Thus, recognizing and employing prompt, trustworthy, and cost-effective approaches like FTIR spectroscopy and data mining techniques become exceedingly crucial for safeguarding public health and ensuring food safety. Therefore, future studies should focus on exploring the specificity of this band when inducing various types of microorganisms into the VBNC state.

Whether or not they enter the VBNC state, microorganisms use various strategies to handle environmental stress. One of these mechanisms involves an increase in RNA synthesis and expression. Especially, under stress conditions, the expression of specific genes may increase, leading to an increase in RNA levels. Furthermore, it is a known fact that microorganisms tend to reduce their metabolic activities to survive for long periods under stress conditions. In this case, bacteria employ energy conservation strategies to maintain their basic cellular functions. Thus, the increased amount of RNA can be an adaptation mechanism for cells to protect their genetic material and potentially reproduce when favorable conditions are reensured (Rhodius et al. 2006; Blazewicz et al. 2013). Studies have shown that dormant organisms contain measurable amounts of rRNA (Chambon et al., 1968), and in some cases, they can contain significantly more rRNA than in the vegetative state (Sukenik et al. 2012). Another reason for the increase in RNA content is the signal transduction system found in microorganisms. In this system, there is a sensor kinase that detects environmental stresses and a response regulator that responds to stresses. Through these mechanisms, certain stress-related genes are regulated, and while intracellular responses are created, the production of RNA molecules that regulate gene expression also increases. (Beier and Gross 2006; Groisman 2016; Piattelli et al. 2020). Furthermore, an increase in the amount of RNA is generally observed during spore formation in microorganisms. The spore formation process means that microorganisms temporarily transit into a resistant form against unsuitable environmental conditions. During this process, the bacterial cell essentially preserves its chromosomal DNA while greatly reducing or stopping its metabolic activities such as nucleic acid synthesis and protein production. However, the spore formation process can lead to the activation of many regulatory genes and increased expression of certain genes. This enables the synthesis of specific proteins that are especially necessary for spore formation. Therefore, during spore formation, there may be an increase in the amount of RNA due to an increase in the expression of certain genes (Segev et al. 2012; Traag et al. 2013; Setlow and Christie 2020). The same applies to biofilm formation, where microorganisms come together to protect themselves from environmental stresses. During biofilm formation, microorganisms adapt to environmental conditions by altering gene expression and metabolic activities. In particular, there is an increase in RNA synthesis and expression by upregulating the expression of various genes to ensure communication and coordination between cells within the biofilm (Ghaz-Jahanian et al. 2013; Martínez and Vadyvaloo 2014; Mitra and Mukhopadhyay 2023; Condinho et al. 2023). Consequently, during the adaptation of bacteria to environmental stresses, the increase in the amount of RNA may be part of their strategy to preserve genetic material and potentially preserve their ability to reproduce. This may be an important adaptation mechanism for bacteria to survive for long periods and adapt to stress conditions while in the VBNC state.

## Conclusion

The findings of the study suggest a complex interplay of biomolecular changes during the induction of the VBNC state, with specific alterations in RNA, protein, and nucleic acid concentrations. The consistency of the findings across different stress conditions, as validated by ROC analysis, reinforces the reliability of the proposed RNA band at 995 cm^− 1^ as a robust biomarker for *E. coli* W3110 in VBNC status. The observed reduction in protein and nucleic acid amounts under certain stress conditions hints at the dynamic nature of cellular responses to environmental stressors, further emphasizing the need for a comprehensive understanding of VBNC-associated modulations. Overall, the combination of chemometric analysis, band integration quantification, and ROC validation strengthens the credibility of the identified spectrochemical biomarker and provides a nuanced perspective on the quantitative changes in biomolecular constituents during the VBNC state induced by different stress factors in *E. coli*. These findings contribute to the advancement of our understanding of bacterial physiological responses and may have practical implications in the rapid detection of VBNC states in laboratory settings. The ability to detect and understand VBNC cells has also serious implications for public health, food safety, environmental monitoring, and microbial control. In conclusion, the application of IR spectroscopy coupled with chemometric analysis proves to be a powerful tool for characterizing and differentiating VBNC states in *E. coli* W3110 under diverse stress conditions. The identified spectral discriminators offer a molecular-level understanding of the biomolecular modulations associated with the VBNC state, contributing to our broader comprehension of bacterial responses to environmental stressors.

## Data Availability

No datasets were generated or analysed during the current study.
